# Functional hyperpolarized gas magnetic resonance imaging of idiopathic pulmonary fibrosis: a systematic review

**DOI:** 10.1007/s11547-026-02256-3

**Published:** 2026-07-07

**Authors:** David Zaccagnini, Andrea Magnini, Lorenzo Cinci, Linda Calistri, Diletta Cozzi, Nicholas Landini, Vittorio Miele, Cosimo Nardi

**Affiliations:** 1 Department of Experimental and Clinical Biomedical Sciences, University of Florence, Largo Brambilla 3, 50134 Florence, Italy; 2 Radiology Emergency Department, Careggi University Hospital, 50139 Florence, Italy; 3 Department of Radiological, Oncological and Pathological Sciences, Policlinico Umberto I Hospital, “Sapienza” Rome University, Rome, Italy

**Keywords:** Magnetic resonance imaging, Interstitial lung disease, Idiopathic pulmonary fibrosis, Usual interstitial pneumonia, Hyperpolarized 129Xeno, Hyperpolarized 3Helium

## Abstract

**Objectives:**

Idiopathic pulmonary fibrosis (IPF) is the most common interstitial lung disease. New non-invasive tools to monitor pulmonary function, disease progression, and therapeutic response are needed. The study was aimed to investigate the role of hyperpolarized 3He and 129Xe MRI in IPF compared with pulmonary function tests and CT.

**Methods:**

A systematic literature review was performed in accordance with the PRISMA guidelines. A specific question based on PICO method was formulated to develop the search strategy, which was limited to English language articles focused on functional hyperpolarized gas MRI on patients affected by IPF. Exclusion criteria were represented by preclinical studies, studies on animals, studies on explanted lungs, conference abstracts, reviews, and case reports. Search was conducted up to September 30, 2025, via PubMed, Embase, Web of Science, and Scopus databases. Data extracted included gas volume inhaled, MRI metrics and sequences, CT imaging comparison, and pulmonary function tests comparison. QUADAS-2 and QUADAS-C were used to assess the risk of bias.

**Results:**

The inclusion criteria were met by 19 studies, which analyzed a minimum of 3 and a maximum of 34 patients. 129Xe was the most frequently employed gas. No defined MRI protocol emerged from the selected studies. A comparison between CT and hyperpolarized gas MRI showed conflicting results, whereas good correlations were observed between hyperpolarized gas MRI, spirometry, and DLco parameters. For example, the correlation between RBC to barrier and DLco was particularly strong (*R* = 0.94, *p* < 0.01). Finally, an overall medium/low risk of bias was found.

**Conclusions:**

Hyperpolarized gas MRI was a potentially promising technique to assess disease progression, response to therapy and prognosis. Nevertheless, such technique needed standardized longitudinal multicenter clinical trials and larger samples to define imaging protocols and to test its capability in detecting IPF changes over time. CT must still be considered crucial in the diagnosis of IPF since typical hyperpolarized gas MRI metrics of IPF remain to be defined at present.

## Introduction

Idiopathic pulmonary fibrosis (IPF) is the most common chronic progressive fibrosing interstitial lung disease (ILD). It is characterized by interstitial matrix deposition with scarring and interstitial thickening, chronic progression, poor prognosis—median survival of 3–5 years—and absence of a certain cause [1–4]. IPF incidence ranges from 3 to 9 cases per 100,000 individuals per year in Europe and North America, and from 1 to 4 cases per 100,000 individuals per year in Asia [5]. Diagnostic tools typically include pulmonary function tests, while computed tomography (CT) is the main imaging technique for the assessment of IPF allowing a confident diagnosis in over 80% of cases [6–9]. Specifically, diagnostic criteria for IPF are represented by the exclusion of other known causes of ILD, the presence of a usual interstitial pneumonia pattern on CT in patients not subjected to surgical lung biopsy and a specific combination of lung tissue and CT patterns in patients subjected to surgical lung biopsy [4].

Current difficulty in standardizing diffusing capacity of the lungs for carbon monoxide (DLco) in multicenter trials, lack of agreement for values of forced vital capacity, and CT inability in detecting disease progression and therapeutic response, make IPF diagnosis and management challenging [10–13]. In addition, management of IPF is even harder due to the alternation of acute exacerbations, chronic stability, and subtle inflammation [1, 2, 10]. The need of new tools to measure pulmonary function and disease progression makes magnetic resonance imaging (MRI) a radiation-free alternative to CT in many diseases, such as cystic fibrosis, pulmonary embolism, and pneumonia [14]. High correlation between MRI and pulmonary function were proved in many studies [15–19]. Furthermore, although limited in characterizing morphology and disease severity because of its low spatial resolution, MRI can provide functional information about treatment response and prognosis, exploiting its capability in detecting water which is a molecule naturally present in inflammation, alveolar infiltrates, and tissue remodeling [14, 16, 17]. Reduction in the number of phase encoding steps and faster MRI sequences, such as Ultrashort Echo Time sequence, have proven to be useful strategies to improve MRI tolerability/effectiveness and to limit signal decay [20, 21].

Hyperpolarized 3Helium (HP 3He) and hyperpolarized 129Xeno (HP 129Xe) MRI were considered to be MRI techniques capable of assessing regional pulmonary structure, function, and gas exchange [22–24]. While low availability and low economic sustainability of 3He gas have greatly limited its diffusion [25], HP 129Xe was particularly remarkable due to its availability, high solubility, and wide chemical-shift range [26, 27]. HP129Xe MRI permitted ventilation imaging since 129Xe gas had resonance frequency shifts in each pulmonary compartment that enabled to use diffusion mapping [28–33]. HP 129Xe MRI was also proved to be a safe, well tolerated, and rapid technique [22–24] able to provide structural and functional information in fibrotic lungs [29, 34, 35]. In addition, a correlation between HP 129Xe MRI parameters and DLco indicated that both techniques investigated the same pathophysiologic mechanisms [29, 36]. Therefore, HP gas MRI could be proposed as a fast and safe imaging technique for structural and functional evaluation of lung parenchyma, especially in IPF that is a pathologic condition in which evidence of disease progression and response to new treatments come still late.

The aim of this review was to perform a systematic analysis on the role of HP gas MRI in IPF compared with pulmonary function tests and CT.

## Materials and methods

### PICO question

This systematic review was conducted following the PRISMA guidelines (Preferred Reporting Items for Systematic reviews and Meta-Analyses). The PRISMA statement is made of a 27-item checklist and a four-phase flow diagram. Its checklist includes items for transparent reporting of a systematic review [37].

PICO is a method commonly used for developing a research question and bibliographic research [38].

Our question was set as follows:

- (P) In patients with diagnosis of IPF
- (I) can HP gas MRI
- (C) using pulmonary function tests and CT imaging as the reference standards
- (O) help us assess disease progression and prognosis?

### Literature searches and inclusion and exclusion criteria

Our search was conducted up to September 30, 2025, via PubMed, Embase, Web of Science, and Scopus databases. The electronic search was carried out using a series of keywords including “magnetic resonance imaging”, “MRI”, “MR”, “lung”, “pulmonary”, “idiopathic pulmonary fibrosis”, “IPF”, “ILD”, “interstitial”, “interstitial lung disease”, “infiltrative”, “xeno”, “xenon”, “helium”, “hyperpolarized gas”.

Search terms were composed using the Boolean operator “OR” to group the terms related to HP gas MRI. The Boolean operator “OR” was also used for the terms indicating the investigated anatomical areas as well as for those indicating the pathological alterations. Then, these three groups of terms were composed using the Boolean operator “AND” to obtain a cross-referenced search between the HP gas MRI imaging technique, the pulmonary anatomical area, and the population affected by IPF (Table 1).

**Table 1:** Search queries used for the identification of studies on Pubmed, Web of Science, Embase, and Scopus databases

PubMed	Embase	Scopus	Web of Science
(((magnetic[Title]) OR (mr[Title]) OR (mri[Title]) OR (xeno[Title]) OR (xenon[Title]) OR (helium[Title]) OR (129 xenon[Title]) OR (129 xeno[Title]) OR (129xe[Title]) OR (129 xe[Title]) OR (3 helium[Title]) OR (hyperpolarized gas[Title])) AND ((pulmonary[Title]) OR (lung[Title])) AND ((interstitial[Title]) OR (infiltrative[Title]) OR (ild[Title]) OR (idiopathic pulmonary fibrosis[Title])))	(‘mri’/exp OR mri OR (magnetic AND (‘resonance’/exp OR resonance))) AND (‘129xe’/exp OR 129xe OR ‘129 xeno’ OR ‘129 xenon’ OR ‘hyperpolarized gas’ OR ‘3 helium’) AND (ipf OR ild OR (idiopathic AND pulmonary AND (‘fibrosis’/exp OR fibrosis)) OR (interstitial AND (‘lung’/exp OR lung) AND (‘disease’/exp OR disease))) AND (‘article’)	( (mri OR ( magnetic AND resonance)) AND ( 129xe OR ‘129 xeno’ OR ‘129 xenon’ OR‘3 helium’ OR’helium’ OR ‘hyperpolarized gas’)) AND ( ipf OR ( idiopathic AND pulmonary AND fibrosis) OR ild OR infiltrative OR ( interstitial AND lung AND disease))	((ALL = (‘mri’)) OR (ALL = (‘magnetic resonance’))) AND ((ALL = (‘129xe’)) OR (ALL = (‘129 xeno’)) OR (ALL = (‘hyperpolarized 129 xeno’)) OR (ALL = (‘3 helium’)) OR (ALL = (‘129 xenon’)) OR (ALL = (‘hyperpolarized gas’)) OR (ALL = (‘xenon’)) OR (ALL = (‘helium’))) AND ((ALL = (‘ipf’)) OR (ALL = (‘Interstitial lung disease’)) OR (ALL = (‘Usual interstitial pneumonia’)) OR (ALL = (‘infiltrative’)) OR (ALL = (‘ild’)) OR (ALL = (‘idiopathic pulmonary fibrosis’)))

To be analyzed in our systematic review, studies had to meet specific inclusion criteria, specifically we collected papers published in English language in international peer-reviewed journals that were focused on functional HP gas 129Xe MRI and 3He MRI on alive human patients affected by IPF. No restrictions were intentionally imposed on the patient selection method, the diagnostic criteria for IPF, the technical parameters and MRI sequences, or which pulmonary function test to use as a reference standard. Exclusion criteria were represented by papers not written in English language, preclinical studies, studies on animals, studies on explanted lungs, conference abstracts, reviews, and case reports. English language was preferred in order to eliminate potential language bias.

### Study selection and data extraction

Titles and abstracts were independently examined by two reviewers in order to identify, select, and determine their eligibility for inclusion. Manual search was also performed using the references of the articles resulting from the database searches. Duplicates were removed through a manual cross-analysis.

No software systems or tools were used for data extraction and management. In case of disagreement concerning study selection or data extraction, a third reviewer independently expressed the final decision. Data were extracted from each study (Tables 2, 3) as follows: first author, year of publication, population, kind of study, post-processing, CT imaging comparison, pulmonary function tests comparison, statistical methods, magnetic field intensity, gas used with hyperpolarized percentage, gas volume inhaled, breath-hold, polarizer, MRI metrics, and MRI sequences.

**Table 2:** Generic data extracted from the articles included in the current review

First author	Year of publication	Population (patient age)	Study design	Software for post-processing	CT score	Pulmonary function test	Statistic method
Hahn et al. [53]	2023	25 cases (70.1 ± 8.5 years)	Cohort prospective (12-month follow-up)	MATLAB	NA	DLCO, FVC, FEV1	Wilcoxon rank-sum, Wilcoxon signed-rank tests
Kaushik et al. [28]	2016	3 cases—4 healthy controls (64.7 ± 8.7 years)	Case–control prospective (14-month follow-up)	MATLAB, MATHWORKS 3D SLICER	Visual comparison	NA	NA
Mata et al. [41]	2021	11 cases—9 healthy controls (65.0 ± 12.5 years)	Case–control	MATLAB	Cases only	DLCO, FVC, FEV1	Mann–Whitney U-test, Pearson correlation coefficient
Guan et al. [42]	2022	29 cases (11 IPF, 13 CF, 5 COPD)—16 healthy controls (66.0 ± 11.6 years, 24.0 ± 8.7 years, 64.0 ± 11.8 years)	Case–control	MATLAB	NA	DLCO, FVC, FEV1	Tukey’s test post hoc analysis
Bier et al. [43]	2019	9 cases—8 healthy controls (66.1 ± 5.6 years)	Case–control	MATLAB	NA	DLCO, FVC, FEV1	Mann–Whitney U-test
Eaden et al. [52]	2023	34 cases (18.0–80.0 years)	Cohort prospective	CALIPER, MATLAB	CALIPER CT and visual CT fibrosis score	DLCO, FVC, DLCO, FVC, FEV1, KCO	Pearson correlation coefficient, Spearman’s rank correlation, Paired sample t-test, Wilcoxon signed-rank tests
Hahn et al. [44]	2022	22 cases—16 healthy controls (71.0 ± 9 years)	Case–control prospective (12-month follow-up)	ANTS	Visual comparison	DLCO, FVC, FEV1, KCO	Wilcoxon signed-rank tests
Chan et al. [45]	2019	18 cases—11 healthy controls (71.4 ± 5.5 years)	Case–control prospective (12-month follow-up)	MATLAB	Likert scoring	DLCO, FVC, FEV1, KCO	Spearman’s rank correlation, Friedman test, post hoc Dunn test, Bland–Altman analysis
Stewart et al. [29]	2015	8 cases (4 IPF, 4 SSc)—10 healthy controls (35.0–85.0 years)	Case–control	MATLAB	NA	DLCO, FVC, FEV1	Patz analytic model, Moxe analytic model, Manson analytic model
Collier et al. [47]	2021	25 cases—10 healthy controls (NA)	Case–control	MATLAB	Visual comparison	DLCO, FVC, FEV1	Pearson correlation coefficient, Spearman’s rank correlation, ANOVA
Qing et al. [48]	2023	10 cases—10 healthy controls (NA)	Case–control	MATLAB	NA	DLCO, FVC, FEV1	Mann–Whitney U-test, Pearson Correlation coefficient, Krippendorff’s coefficient
Weatherley et al. [54]	2019	18 cases (67.8–74.3 years)	Cohort prospective (12-month follow-up)	MATLAB	NA	DLCO, FVC, FEV1	Spearman’s rank correlation, Friedman test, Bland–Altman analysis
Wang et al. [35]	2017	12 cases—13 healthy controls (66.0 ± 15 years)	Case–control	ANTS	Visual comparison	NA	NA
Wang et al. [34]	2018	12 cases—13 healthy controls (66.0 ± 15 years)	Case–control	NA	NA	DLCO, FVC, FEV1	NA
Tibiletti et al. [49]	2023	41 cases (14IPF, 11 HP, 11.DI-ILD, 5 CTD-ILD)—10 healthy controls (NA)	Case–control prospective (1- to 6-month follow-up)	ANTS	NA	DLCO, FVC, FEV1	Pearson correlation coefficient, Kruskal–Wallis rank test, ANOVA
Kaushik et al. [26]	2014	6 cases -11healthy controls (66.8 ± 12.3 years)	Case–control	MATLAB	NA	DLCO, FVC, FEV1	Wilcoxon Rank-sum test
Robertson et al. [50]	2017	12 cases—12 healthy controls (68.5 ± 7.2 years)	Case–control	MATLAB	Visual comparison	DLCO, FVC, FRC, TC	Mann–Whitney U-test, Wilcoxon Rank-sum test
Kammermann et al. [51]	2020	16 cases—8 healthy controls (69.0 ± 7 years)	Case–control	MATLAB	NA	DLCO, FVC, FEV1	Spearman’s rank correlation, Wilcoxon Rank-Sum
Chan et al. [46]	2023	16 cases—5 healthy controls (71.0 ± 5 years)	Case–control	CALIPER, MATLAB	NA	DLCO, FVC, FEV1	Bland·-Altman analysis

IPF: Interstitial Pulmonary Fibrosis; CF: Cystic Fibrosis; HP: Hypersensitivity pneumonitis; COPD: Chronic obstructive pulmonary disease; SSC: Systemic Sclerosis; DI-ILD: Drug-Induced Interstitial Lung Disease; CTD-ILD: Interstitial Lung Disease associated with Connective Tissue Disease; DLCO: Diffusing capacity of the lungs for carbon monoxide; FVC: Forced Vital Capacity; FEV1: Forced Expiratory Volume in one second; TC: Total Capacity; FRC: Functional Residual Capacity; KCO: Carbon monoxide transfer Coefficient; NA: not available

**Table 3:** MRI data extracted from the articles included in the current review

First author	Year of publication	Magnetic field strength	Kind of gas	Gas volume and breath-hold duration	Polarizer	Metrics	Sequences
Hahn et al	2023	1.5 T	129Xe	NA—Breath-hold 15s	NA	RBC/gas, Tissue/gas, RBC/membrane ratio, Ventilation defect percentage	3D radial 1-point Dixon
Kaushik et al	2016	1.5 T	129Xe	1L—Breath-hold 13-15s	Polarean, Durham	RBC/gas, Tissue/gas, RBC/tissue, Ventilation defect percentage	3D radial 1-point Dixon
Mata et al	2021	1.5 T	129Xe	1L—Breath-hold < 10s	NA	RBC/gas, Tissue/gas, RBC/tissue	3D Single-Breath Chemical-Shift Imaging Gradient Echo image
Guan et al	2022	1.5 T	129Xe	1L—Breath-hold 7s	NA	RBC/gas, Tissue/gas, RBC/tissue	3D Single-Breath Chemical-Shift Imaging Gradient Echo image
Bier et al	2019	1.5 T	129Xe	1L—Breath-hold 8s	Model 9810, Polarean	Spectrum temporal variation (RBC/barrier ratio, amplitude, spectrum, chemical shift, full width at half maximum)	Spectroscopy with FID sampling
Eaden et al	2023	1.5 T	129Xe	1L—Breath-hold 16s	NA	Regional Xe-ADC, Mean acinar dimension	3D spoiled gradient echo multiple b-value, DWI/ADC
Hahn et al	2022	1.5 T/3T	129Xe	1L—Breath-hold 15s	NA	RBC/gas, Tissue/gas, RBC/tissue, Ventilation defect percentage	3D radial 1-point Dixon
Chan et al	2019	1.5 T	3He	1L—Breath-hold 15s	NA	Xe-ADC, Mean acinar dimension	3D spoiled gradient echo multiple b-value, DWI/ADC
Stewart et al	2015	1.5 T	129Xe	1L—Breath-hold 15s	Spin-exchange optical pumping	Alveolar septal thickness, Capillary transit time, Surface-area/volume	Chemical-shift saturation recovery spectroscopic imaging technique
Collier et al	2021	1.5 T	129Xe	NA—Breath-hold 14s	Spin-exchange optical pumping	RBC/TP (tissue/plasma), RBC/gas, TP/gas	Echo fly back 3 D radial spectroscopic imaging technique
Qing et al	2023	1.5 T	129Xe	1L—Breath-hold 10s	NA	Tissue/gas	NA
Weatherley et al	2019	1.5 T	129Xe	1L—Breath-hold 10-15s	NA	RBC/TP	NA
Wang et al	2017	1.5 T	129Xe	1L—Breath-hold 13-15s	Model 9810, Polarean	RBC/gas, Tissue/gas, RBC/tissue	3D radial 1-point Dixon and 3D radial sequence
Wang et al	2018	1.5 T	129Xe	1L—Breath-hold 13-15s	Model 2881, Polarean	RBC/gas, Tissue/gas, RBC/tissue	3D radial 1-point Dixon
Tibiletti et al	2023	1.5 T	129Xe	1L—NA	Spin-exchange optical pumping	129Xe ventilation map, T1map, OE-MRI, ventilation % /ventilation heterogeneity	3D balanced steady-state free precession, 3D spoiled gradient echo, Inversion recovery T1 mapping
Kaushik et al	2014	1.5 T	129Xe	1L—Breath-hold 4-8s	Model 9800, Polarean	RBC/gas, Tissue/gas, RBC/tissue	NA
Robertson et al	2017	1.5 T	129Xe	1L—Breath-hold 13s	Model 9810, Polarean	RBC/tissue, F I D decomposed into spectral components	NA
Kammermann et al	2020	1.5 T	129Xe	1L—Breath-hold 15s	Model 9820, Polarean	R2*, RBC/gas, Tissue/gas, RBC/tissue	NA
Chan et al	2023	1.5 T	129Xe/3He	1L—NA	NA	Gases ADC, Mean acinar dimension	3D balanced steady-state free precession, 3D spoiled gradient echo multiple b-value, DWI/ADC

RBC: Red Blood Cells; Xe-ADC: Xenon Apparent Diffusion Coefficient; DWI: Diffusion Weighted Imaging; OE-MRI: oxygen-enhanced MRI; FID: Free Induction Decay; L: liter; S: seconds; NA: not available

### Risk of bias

The quality assessment tools QUADAS-2 (Quality Assessment Tool for Diagnostic Accuracy Studies-2) and QUADAS-C (Quality Assessment Tool for Diagnostic Accuracy Studies-C) were used to evaluate the risk of bias.

QUADAS-2 is a tool specifically designed for evaluating risk of bias and applicability in systematic reviews of accuracy studies [39].

QUADAS-C is a tool for systematic reviews of comparative accuracy studies [40], basically an extension to QUADAS-2. QUADAS-C can be used for reviews on diagnostic accuracy addressing comparative accuracy questions. The use of the QUADAS-2 and -C methods offered a complete and effective framework for the assessment of the potential risk of bias of each study included in the current systematic review, ensuring reliability of the conclusions reached.

## Results

### Study selection

Sixty-four, eighty-six, thirty-five, and eighty-eight studies were identified from PubMed, Embase, Scopus, and Web of Science databases, respectively. Seventeen papers were also found from manual search using the bibliographic references of the articles resulting from the database searches. Duplicates were removed. Fifty-four studies were excluded for not meeting the inclusion criteria based on titles and abstracts. Twenty-seven full-text articles were assessed for eligibility, eight of which were excluded because they were not original articles or were not focused on IPF. Nineteen articles were finally selected for evaluation. Specifically, they dealt with functional HP gas MRI in patients affected by IPF. Such studies were published between 2015 and 2025.

**Fig. 1:**
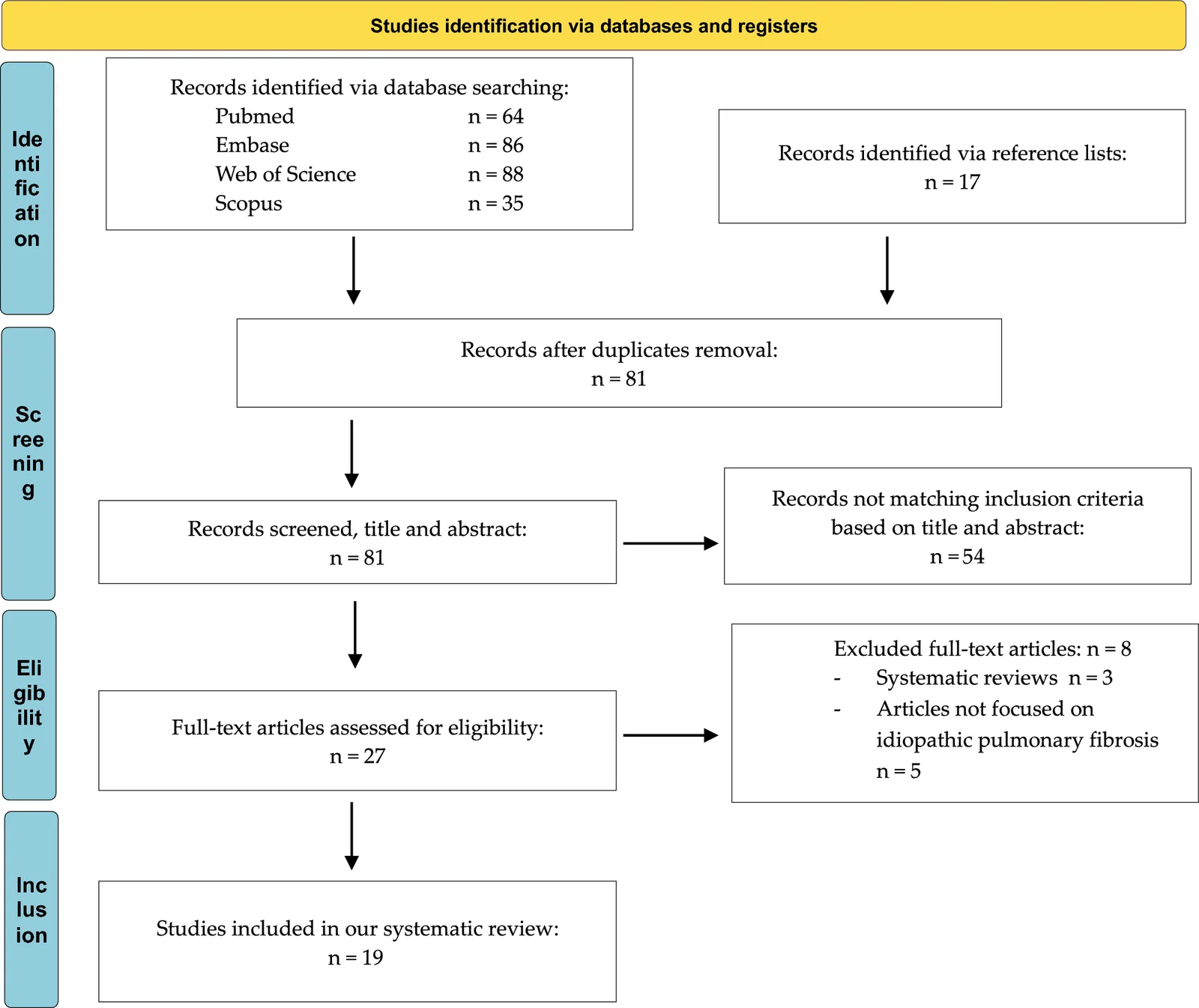
PRISMA statement for systematic review. Flowchart illustrating literature research and papers identification process via databases and registers

The articles selection process was summarized in a flow diagram (Fig. 1).

### Data extraction

*Study design and HP gas* (Table 2). The larger part of the selected papers was designed as case–control study and compared IPF patients with healthy controls [26, 28, 29, 34, 35, 41–51], three of whom compared IPF patients with patients affected by different ILD too [29, 42, 49]. Three papers were designed as a prospective cohort study [52–54]. Unfortunately, the diagnostic pathway for IPF was not specified in any of the articles examined. No information was provided about how and when diagnosis of IPF was made, neither how many diagnostic criteria were satisfied in order to make diagnosis. In addition, no patient medical history was provided.

HP 129Xe was used in all the studies except one in which the gas at issue was HP 3He [45]. In addition, Chan et al. used both HP 129Xe and HP 3He [46], specifically they switched from HP 3He to HP 129Xe because of the high cost of Helium gas.

*Key MRI sequences* (Table 3). No defined imaging protocol emerged from the selected studies. Several strategies were applied to perform chemical-shift separation and gas mapping. The most commonly applied sequences were one-point Dixon technique [28, 34, 35, 44, 53] and 3D spoiled gradient echo [41, 42, 45, 46, 49, 52]. In Stewart et al. study [29], Chemical-Shift Saturation Recovery spectroscopic technique was employed, demonstrating that HP 129Xe spectroscopy can detect alterations in lung structure and function.

*Comparison with CT* (Table 2). MRI and CT were compared in eight studies [28, 35, 41, 44, 45, 47, 50, 52]. Eaden et al. [52] found that 129Xe apparent diffusion coefficient (ADC)-MRI was moderately correlated with a semi-quantitative visual CT fibrosis score—a method used to visually assess the extent of lung fibrosis on CT examinations—in the lung lower zones. Mata et al. [41] observed that regions in which HP 129Xe concentration was higher in interstitial tissue than in red blood cells (RBCs)—high tissue to RBC ratio—corresponded to areas of fibrosis on CT images. In another study [26] HP 129Xe gas transfer defects corresponded to fibrosis located in posterior and anterior lung areas on CT images, whereas 28% of the 129Xe-RBC defects were located in areas where CT imaging did not show alterations, probably because of subtle inflammation, barrier thickening, and impaired gas transfer. On the contrary, Wang et al. [34] proved that HP 129Xe MRI metrics correlated poorly and insignificantly with visual CT fibrosis scores, probably due to the limitation of the reader-based methodology or to the high sensitivity of HP gas MRI to detect fibrotic alterations of regional gas exchange at the alveolar level.

*Comparison with pulmonary function tests* (Table 2). A comparison between MRI metrics, traditional spirometry parameters, and DLco was performed in almost all the studies selected. According to Kaushik et al. [28], a good correlation between global RBC to tissue ratio and DLco was found (*R* = 0.89, *p* < 0.0001), whereas Mata et al. [41] proved good correlation between tissue to RBC ratio and forced vital capacity (*R* =  − 0.78, *p* < 0.01) and between tissue to RBC ratio and DLco (*R* =  − 0.85, *p* < 0.01). In Wang et al. study [34], HP 129Xe gas exchange imaging metrics significantly correlated with both forced vital capacity and DLco when averaged over the whole lungs. In particular, the correlation between RBC to barrier and DLCO was particularly strong (*R* = 0.94, *p* < 0.01). Finally, HP 129Xe ADC-MRI had strong correlation with DLco and carbon monoxide transfer coefficient (*R* =  − 0.65; *p* < 0.001) [52].

### Risk of bias

An overall medium/low risk of bias was found based on the parameters of the QUADAS-2 and QUADAS-C tools (Table 4).

**Table 4:**
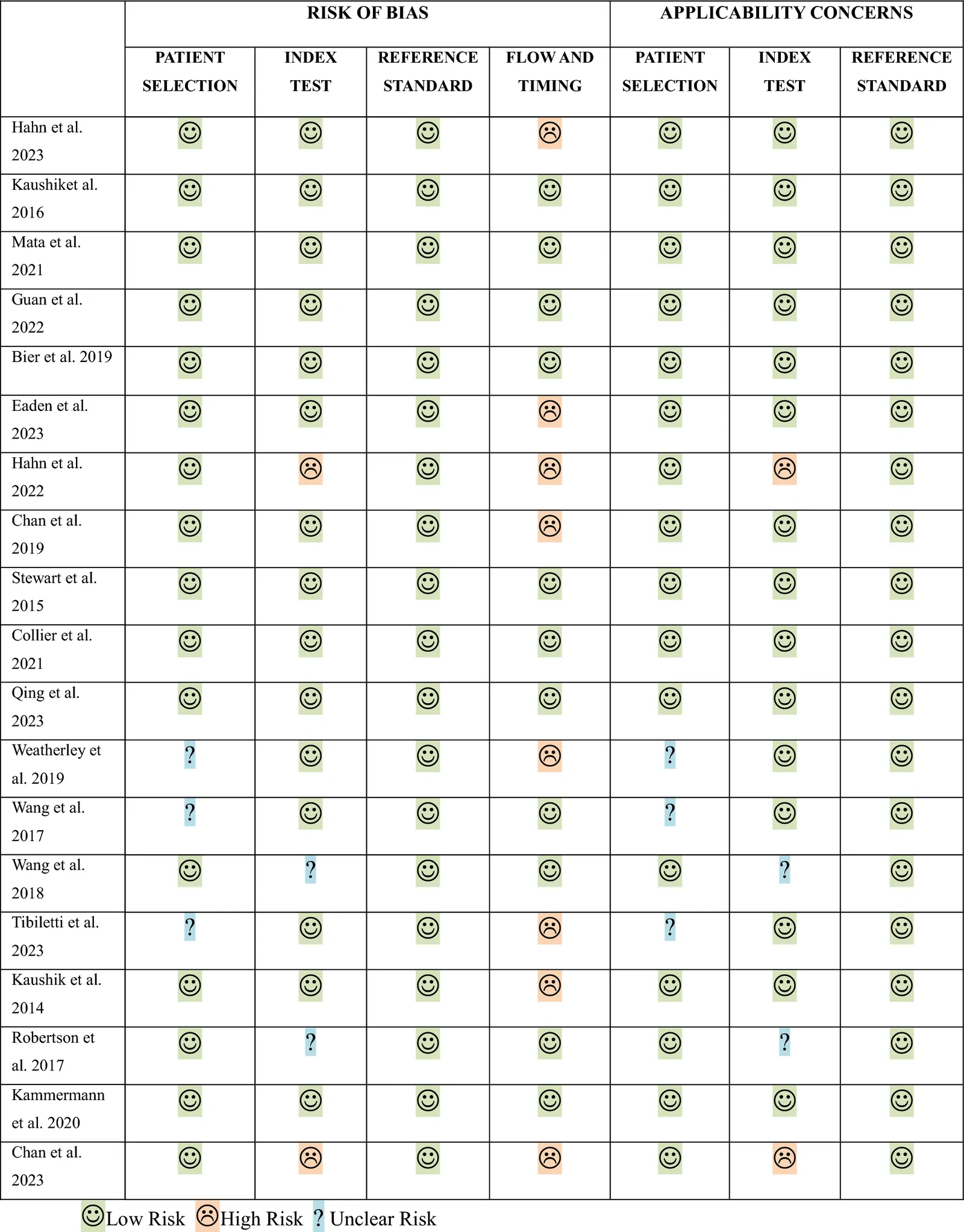
Estimated risk of bias of the included studies (QUADAS-2 and QUADAS-C)

Most of the results with high risk of bias were observed in the flow and timing column due to the absence of standardized follow-up timing and loss of patients. Specifically, cases and controls underwent different follow-up examinations [45, 46] or follow-up examinations were delayed because of COVID-19 pandemic restrictions [52]. Loss of patients was mainly due to the lack of tolerability to MRI examinations or death [44, 45, 49, 52, 53]. Absence of standardized follow-up timing was also noticed in Kaushik et al. [26] because only nine out of eleven healthy volunteers underwent a follow-up examination and no IPF patient underwent a follow-up examination.

Recurrent limits in all studies were small IPF cohorts (three to thirty-four patients) and the partial comparability of cases and controls because of age difference between patients affected by IPF and healthy cohorts, being controls younger.

In the index text column we considered two cases [34, 50] with unclear risk of bias in which the gas inhaled was not proportional to patients’ lung volumes and also two cases with high risk of bias because of switching from 1.5T to 3T MRI-units [44] and from HP 3He to HP 129Xe [46].

In the patient selection column, unclear risk of bias was associated to comorbidities that could have impacted study results. For example, unclear risk of bias was associated to the non-uniform distribution of patients across the diseased groups and to different treatment regimens before and after recruitments [49] or IPF cohort included five patients that were already taking antifibrotic treatment, whereas five different patients started therapy during the ongoing study [54].

Finally, low applicability concerns were found, precisely three cases of unclear risk of bias in patients’ selection [35, 49, 54] and two cases both of unclear risk of bias [34, 50] and high risk of bias [44, 46] in the index text.

## Discussion

### Preliminary considerations

HP gas MRI represented a potentially useful technique to evaluate the regional functional modifications in IPF. Nevertheless, CT imaging remains the gold standard technique in making the diagnosis of IPF, being able to prove usual interstitial pneumonia (UIP) pattern of fibrosis characterized by honeycombing cysts, reticular septal thickening, and traction bronchiectasis with subpleural and posterior basal predominance [55]. CT is known to be a fast, well tolerated and widely available technique. However, it lacks functional information and has low sensitivity for fibrosis progression and therapeutic response [6, 8, 12, 19]. In addition, matrix microdeposition and tissue thickening are not directly detectable on both CT and morphological MRI [14, 16, 56]. On the contrary, despite low spatial resolution, HP gas MRI showed an encouraging sensitivity in detecting functional changes and it was proposed as a good candidate for the assessment of respiratory function, prognosis, and therapeutic response [22, 24, 26, 57]. The good sensitivity of HP 129Xe MRI to microstructural changes was also demonstrated by high HP gas ADC-MRI and mean diffusive length scale values—an estimate of the acinar airway size calculated via gas distribution—in areas showing fibrotic pattern on CT images [52, 58]. HP 3He MRI exhibited high-resolution for lung diffusion imaging enabling pulmonary ventilation color mapping with a view to show different signal intensity in different lung portions and to reveal ventilation dynamics and defects [59]. In fact, HP 3He MRI may be used not only for IPF, but also in the investigation of chronic obstructive pulmonary disease, asthma, cystic fibrosis, and ILDs [45, 46, 56, 60]. Unfortunately, the use of HP 3He was not economically sustainable due to the low availability and high cost of Helium gas, namely 2400$–3000$ per liter versus 30$ per liter for Xenon gas [61, 62]. For this reason, Chan et al. switched from Helium to Xenon [46] after having used HP 3He only in a previous study [45]. On the contrary, the spread of HP 129Xe has been increasing in the last few years because of its low cost, high availability and chemical-shift properties. This was the reasons why HP 129Xe gas largely predominated in the selected papers of this review.

### Technical considerations on HP gas and MRI sequences

Typical Xenon gas mixture for MRI is commonly made of Xenon and Nitrogen, has 1-L volume and contains 20–50% Xenon polarization content [26, 29, 34, 35, 41, 42, 46, 49, 50]. Hyperpolarization is generally obtained through the spin exchange optical pumping technique, giving Xenon a spin ½ nucleus in order to make it detectable on MRI [27]. After inhalation, HP 129Xe physiologically passes through the alveolar-capillary barrier membrane into the capillary networks and enters into the RBCs. It is known that about 2% of inhaled HP 129Xe dissolves into pulmonary tissue, plasma and RBCs, each of which shows a specific spectral peak that can be used to detect every phase of gas dissolution [48, 54, 63]. Thusly, it was possible to reconstruct ventilation dynamics with MRI diffusion color mapping through the detection of different frequency shifts in airways, interstitial compartment, and RBCs [26, 48, 64].

Three main ratio measures of normalized gas exchanges emerged from the selected papers, namely RBC to gas, tissue to gas, and RBC to tissue ratios. Such signal ratios were expression of different gas concentrations in each lung compartment and showed good correlation with conventional physiologic measurements of gas exchange, reflecting gas entrapment and diffusion impairment due to the histological modifications of lung tissues, especially in later stages of IPF [26]. Kaushik et al. [26] acquired the spectroscopic HP 129Xe signal in each lung compartment and measured the simple ratio of HP 129Xe signal concentrations in both RBCs and interstitial tissue. They found that HP 129Xe signal in RBCs, quantified in RBC to tissue ratio, was 3.3-fold lower in IPF patients than in healthy controls. Tissue to RBCs ratio resulted as a probe of gas transfer effectiveness and HP 129Xe lung distribution ratios well correlated with DLco.

Several MRI expedients were used to achieve HP 129Xe chemical-shift separation, nevertheless specific imaging protocols have not still been established.

The one-point Dixon method has been historically one of the favored approaches to produce MRI quantitative maps and offered an effective way to image barrier uptake and RBC transfer [34, 35]. It employed a submillisecond echo-time that mitigated the impact of short HP129Xe T2*-MRI in the dissolved phase. This method was subsequently modified and paired with a second and longer echo-time to carry out gas-phase signal examination and to delete residual gas signal contamination of a similar order of magnitude [50]. The one-point Dixon method had image blurring as main limitation due to the mixing of the distinct compartment signals away from the center of K-space [51], which in technical terms is an array of numbers representing spatial frequencies in the MR image.

Another chemical-shift separation technique was the hierarchical iterative decomposition of water and fat with echo asymmetry and least squares estimation (IDEAL) [65]. To enhance sensitivity to regional disease, Qing et al. [33] used IDEAL technique in conjunction with simple ratios of HP 129Xe signal. IDEAL reconstruction was applied to HP 129Xe dissolved-phase images with spiral trajectory to quantify Xenon gas transfer kinetics at several repetition times and to carry out chemical-shift saturation recovery imaging [28, 50]. Kammerman et al. [51] suggested a comprehensive model-based reconstruction of the IDEAL method by a four-point spectroscopic imaging technique incorporating T2* values, field inhomogeneity maps and frequency shifts. They proved that the transverse relaxation rate of dissolved-phase HP 129Xe may be a biomarker of lung injury in IPF.

An implementation of echo-planar spectroscopic imaging radial-trajectory pulse sequence consisted of a system that captured multi-echo HP 129Xe dissolved-phase and gas-phase images over the whole lung in a short breath-hold, achieving the chemical-shift modeling and separation in K-space [47]. Finally, 3D Single-breath Chemical-Shift Imaging (3D-SBCSI) was developed to detect and measure HP 129Xe spectral parameters [42]. 3D-SBCSI was able to generate the full spectrum of peaks per voxel, helping to discriminate different lung diseases since it proved to be sensitive to the physiologic changes caused by the pulmonary abnormalities associated to ventilation defects, tissue T2* relaxation, and RBC frequency shift [41, 42].

### Correlation with CT and pulmonary function tests

HP gas MRI capability in the detection of early functional alterations was overall relevant compared to CT. While in Wang et al. study [35] the correlation between MRI and CT was not significant, Guan et al. [42] observed that patients with IPF had severely reduced RBC uptake in areas of fibrosis detected on CT, thus proving the correlation between fibrosis and gas transfer impairment. In addition, some authors [28, 44, 46, 52] found DW-MRI to be sensitive to early microstructural changes in lung interstitium and capable of revealing abnormalities even in lung areas that were designed as normal on CT imaging, suggesting that DW-MRI might play a crucial role in the development of reliable imaging biomarkers to monitor IPF progression and response to treatment.

According to Hahn et al. [44], the extent of fibrosis on CT and functional measures of gas exchange or ventilation on MRI were associated with disease progression in IPF one year later. Such evidence suggested that the high sensitivity of MRI to pulmonary microstructural and functional changes made it capable of examining longitudinal functional modifications, but it did not allow to perform initial diagnosis of IPF because of the poor signal-to-noise ratio and absence of typical IPF metrics. HP gas MRI sensitivity still needed to be proven in patients with earlier disease, even in patients without the typical usual interstitial pneumonia pattern on CT images. A further interesting parameter that might play a role in monitoring disease progression was R2*-MRI for barrier and RBC compartments [51], indicated as a potential biomarker of lung injury, but still needing a defined reconstruction methodology to estimate its regional variations.

The papers examined in the current review reported an overall satisfactory correlation between HP 129Xe distribution ratios and pulmonary function tests. A good correlation was demonstrated between RBC to tissue ratio and both DLco and forced vital capacity [35, 41, 48]. A good correlation was also found between 129Xe ADC-MRI and both DLco and carbon monoxide transfer coefficient [52]. In addition, Guan et al. [42] observed that tissue to RBC ratio and RBC Chemical Shift in 3D-SBCSI sequences appeared to be even more sensitive parameters than traditional spirometry, being responsive to subtle alterations in lung structure.

### Limitations and future directions

Our analysis on HP gas MRI techniques might have been influenced by some potential sources of bias (Table 4). A total lack of standardized follow-up timing and standardized outcome measures across studies was found. Data emerged from the selected papers were mostly recorded at baseline examination and needed to be monitored in longitudinal cohort studies to develop a technique able to monitor disease progression. A strictly planned follow-up timing will also allow to investigate disease evolution and identify subsets of patients with different response to therapy.

Even though mostly studies stated that HP gas MRI was an efficient technique to assess pulmonary function, they were limited by a small number of IPF patients ranging from three to thirty-four.

Furthermore, typical IPF metrics must still be identified to unequivocally detect such disease. In Tibiletti et al. study [49], ventilation-only biomarkers were not suitable to recognize different ILD. Preliminary evidence suggested that some pulmonary vascular diseases were characterized by enhanced barrier and reduced RBC to tissue ratios [35, 66]. Non-specific interstitial pneumonia was characterized by enhanced barrier uptake and both regionally enhanced and deficient RBC transfer [67]. Developing additional biomarkers and finding a distinct MRI profile of IPF will be a crucial future challenge.

Another potential source of bias was the presence of comorbidities and different timing of therapy in IPF cohorts. These factors influenced pulmonary and cardiac functions affecting diagnostic capacity in the assessment of gas diffusion, with consequent partial or ambiguous results. IPF metrics should be evaluated in patient without such confounding factors.

In addition, the death of some patients and the loss of breath-hold tolerability in dyspnoeic patients demanded attention as a source of bias in small cohorts. A high risk of impairment of the obtained results was also associated to switching from 1.5T to 3T examinations and switching from 3He to 129Xe, as found in two papers [45, 46].

Given the several strategies to image gases in lungs via different MRI-units and the wide choice of MRI techniques experimented to identify HP 3He and HP 129Xe in each lung compartments, future trials will be required to design both a definitive imaging protocol and a standard post-processing protocol in order to define the best sequences to quantify HP gas metrics. In fact, despite HP 129Xe metrics were measured with high reproducibility, they depended somewhat on repetition times and flip angles used for MRI acquisitions. Moreover, being MRI-generated images inherently qualitative—no numerical value—further processing will be needed to create quantitative 3D maps to discriminate HP 129Xe dissolved phases.

In light of the aforementioned limitations and correlations between HP gas MRI and CT/pulmonary function tests, ideal prospective case–control studies should include large groups of patients, comparable healthy controls of the same age range, no comorbidity in cases and controls cohorts, standardized follow-up timing, same timing of antifibrotic therapy or no antifibrotic therapy, same HP gas, and MRI-units with similar parameters and sequences. Therefore, taking into account what was above mentioned, the encouraging results described in the current review has to be considered with caution, waiting for further confirmation.

## Conclusions

This systematic review proved that HP gas MRI was a potentially promising technique to assess functional modifications in IPF. While CT must be still considered the cornerstone for IPF diagnosis and disease monitoring, HP gas MRI exhibited good correlation with pulmonary function tests. HP gas MRI capability in the detection of pulmonary microstructural and functional changes, even before morphologic alterations on CT imaging, showed significant potential for further development in monitoring IPF progression, response to therapy, and prognosis. Current studies on the role of functional HP gas MRI of IPF suggested that HP 129Xe MRI may evince regional ventilation and gas exchange abnormalities and that its correlation with DLCO or FVC was overall good. Finally, the prognostic value and clinical standardization of these metrics remained to be established. In fact, a standardized imaging protocol was missing and longitudinal multicenter clinical trials with large sample size will be needed to define appropriate imaging protocols and to test HP gas MRI capability in the detection of IPF changes over time.
